# Left Hemisphere Bias of NIH Stroke Scale Is Most Severe for Middle Cerebral Artery Strokes

**DOI:** 10.3389/fneur.2022.912782

**Published:** 2022-06-14

**Authors:** Emilia Vitti, Ganghyun Kim, Melissa D. Stockbridge, Argye E. Hillis, Andreia V. Faria

**Affiliations:** ^1^Department of Neurology, School of Medicine, Johns Hopkins University, Baltimore, MD, United States; ^2^Department of Neuroscience, Johns Hopkins University, Baltimore, MD, United States; ^3^Department of Physical Medicine, Rehabilitation, and Cognitive Science, Johns Hopkins University, Baltimore, MD, United States; ^4^Department of Radiology, School of Medicine, Johns Hopkins University, Baltimore, MD, United States

**Keywords:** stroke, NIHSS, MCA, bias, left hemisphere

## Abstract

NIHSS score is higher for left vs. right hemisphere strokes of equal volumes. However, differences in each vascular territory have not been evaluated yet. We hypothesized that left vs. right differences are driven by the middle cerebral artery (MCA) territory, and there is no difference between hemispheres for other vascular territories. This study is based on data from 802 patients with evidence of acute ischemic stroke in one major arterial territory (MCA, *n* = 437; PCA, *n* = 209; ACA, *n* = 21; vertebrobasilar, *n* = 46). We examined differences in patients with left or right strokes regarding to lesion volume, NIHSS, and other covariates (age, sex, race). We used linear models to test the effects of these covariates on NIHSS. We looked at the whole sample as well as in the sample stratified by NIHSS (≤5 or >5) and by lesion location (MCA or PCA). Patients with left MCA strokes had significantly higher NIHSS than those with right strokes. Only patients with MCA strokes showed NIHSS score affected by the hemisphere when controlling for stroke volume and patient's age. This difference was driven by the more severe strokes (NIHSS>5). It is important to consider this systematic bias in the NIHSS when using the score for inclusion criteria for treatment or trials. Patients with right MCA stroke may be under-treated and left with disabling deficits that are not captured by the NIHSS.

## 1. Introduction

The National Institutes of Health Stroke Scale (NIHSS) is a valid and reliable tool most frequently used for clinically evaluating acute stroke (1–4). The NIHSS is associated with severity, long-term functional outcomes (5–7), infarct size, lesion location (1), and angiographic findings (8–10). Scoring the NIHSS consists of broad categories associated with stroke signs and symptoms (e.g., level of consciousness, motor performance, language, speech, neglect, etc.). Distinct clinical features or stroke syndromes can be appreciated depending on the specific vascular territory affected [i.e., anterior cerebral artery (ACA), posterior cerebral artery (PCA), middle cerebral artery (MCA)].

The NIHSS is designed to represent left and right cortical and motor function equally; however, there are more opportunities to award points for left hemisphere dysfunction than right (8, 11, 12). This is likely because up to 7 points are directly related to language deficits, and these deficits typically are associated with left MCA stroke only, particularly among right-handed people. Points attributable to left MCA cortical strokes are awarded across three categories: (1) orientation questions, (2) following commands, and (3) specific language tasks to determine signs of aphasia (e.g., picture description, confrontation naming, sentence reading), in addition to sensory and motor. In contrast, points attributable to right MCA cortical stroke are awarded in only one category: (1) neglect, (2) other than sensory and motor (Supplementary Table 1). Thus, even if the stroke volume is equal, NIHSS scores are often higher for left vs. right hemisphere stroke (8, 11). This bias may partially account for findings that stroke patients with right-hemisphere infarcts are 45% less likely to be treated with thrombolysis than patients with left-hemisphere infarcts (13).

**Table 1:** Demographic and lesion characteristics.

	**All**			**MCA**			**PCA**		
	**Total (*N* = 802)**	**Left (*N* = 420)**	**Right (*N* = 382)**	**Total (*N* = 498)**	**Left (*N* = 251)**	**Right (*N* = 247)**	**Total (*N* = 237)**	**Left (*N* = 124)**	**Right (*N* = 113)**
NIHSS	5.5 (5.9) [0–31]	5.8 (6.4) [0–31]	5.1 (5.4) [0–24]	6.9 (6.7) [0–31]	7.5^*^ (7.2) [0–31]	6.3^*^ (6.1) [0–24]	3.0 (2.8) [0–19]	3.1 (3.1) [0–19]	2.9 (2.4) [0–11]
lesion volume (cc)	23.8 (52.2)	22.8 (513)	25.02 (53.2)	34.66 (62.9)	33.93 (63.8)	35.41 (63.1)	6.8 (14.9)	7.2 (16.9)	6.4 (12.6)
	[0.028–403.4]	[0.028–383.4]	[0.042–403.46]	[0.06–403.46]	[0.084–383.4]	[0.06–403.46]	[0.028–131.6]	[0.028–131.6]	[0.042–73.7]
Age	62 (13)	61 (13)	62 (14)	62 (14)	62 (13)	62 (15)	62 (12)	60 (13)^*^	63 (12)^*^
Sex									
Female	370 (46.1%)	191 (45.5%)	179 (46.9%)	233 (46.8%)	118 (47.0%)	115 (46.6%)	101 (42.6%)	50 (40.3%)	51 (45.1%)
Male	432 (53.9%)	229 (54.5%)	203 (53.1%)	265 (53.2%)	133 (53.0%)	132 (53.4%)	136 (57.4%)	74 (59.7%)	62 (54.9%)
Race									
African American	485 (60.5%)	249 (59.3%)	236 (61.8%)	279 (56.0%)	130 (51.8%)	149 (60.3%)	165 (69.6%)	92 (74.2%)	73 (64.6%)
Caucasian	276 (34.4%)	148 (35.2%)	128 (33.5%)	190 (38.2%)	104 (41.4%)	86 (34.8%)	65 (27.4%)	29 (23.4%)	36 (31.9%)
Other / Unknown	41 (5.1%)	23 (5.5%)	18 (4.7%)	29 (5.8%)	17 (6.8%)	12 (4.9%)	7 (3.0%)	3 (2.4%)	4 (3.5%)
Vascuar Territory									
MCA	498 (62.1%)	251 (59.8%)	247 (64.7%)						
PCA	237 (29.6%)	124 (29.5%)	113 (29.6%)						
Vertebrobasilar	46 (5.7%)	27 (6.4%)	19 (5.0%)						
ACA	21 (2.6%)	18 (4.3%)	3 (0.8%)						

*Continuous variables are showed as mean (standard deviation) [minimum–maximum]*.

* *Indicates significant difference at p < 0.05*.

Differences in NIHSS relative to specific vascular territories have not yet been evaluated. Deficits associated with bilateral PCA and ACA stroke are likely symmetrically represented in the NIHSS scale, because language is largely specific to the left MCA territory vs. right. However, left ACA and PCA stroke can cause aphasia [e.g., transcortical motor aphasia, optic aphasia; see (14)]. Hemispatial neglect can be caused by ACA, MCA, or PCA stroke. While neglect is more noticeable after right hemisphere stroke, right neglect after left hemisphere stroke may be almost as common (15). Thus, there are clear reasons to suspect that hemispheric bias in the NIHSS may be specific to MCA territory strokes. We hypothesized that right MCA strokes have larger infarct volumes than left MCA ischemic strokes, in groups with similar NIHSS, but there will be no difference between hemispheres for other vascular territories. Similarly, we hypothesized that after controlling for stroke volume and other covariates, the side of MCA infarcts, but not of infarcts in other territories, significantly affects the NIHSS.

## 2. Methods

**Figure 1:**
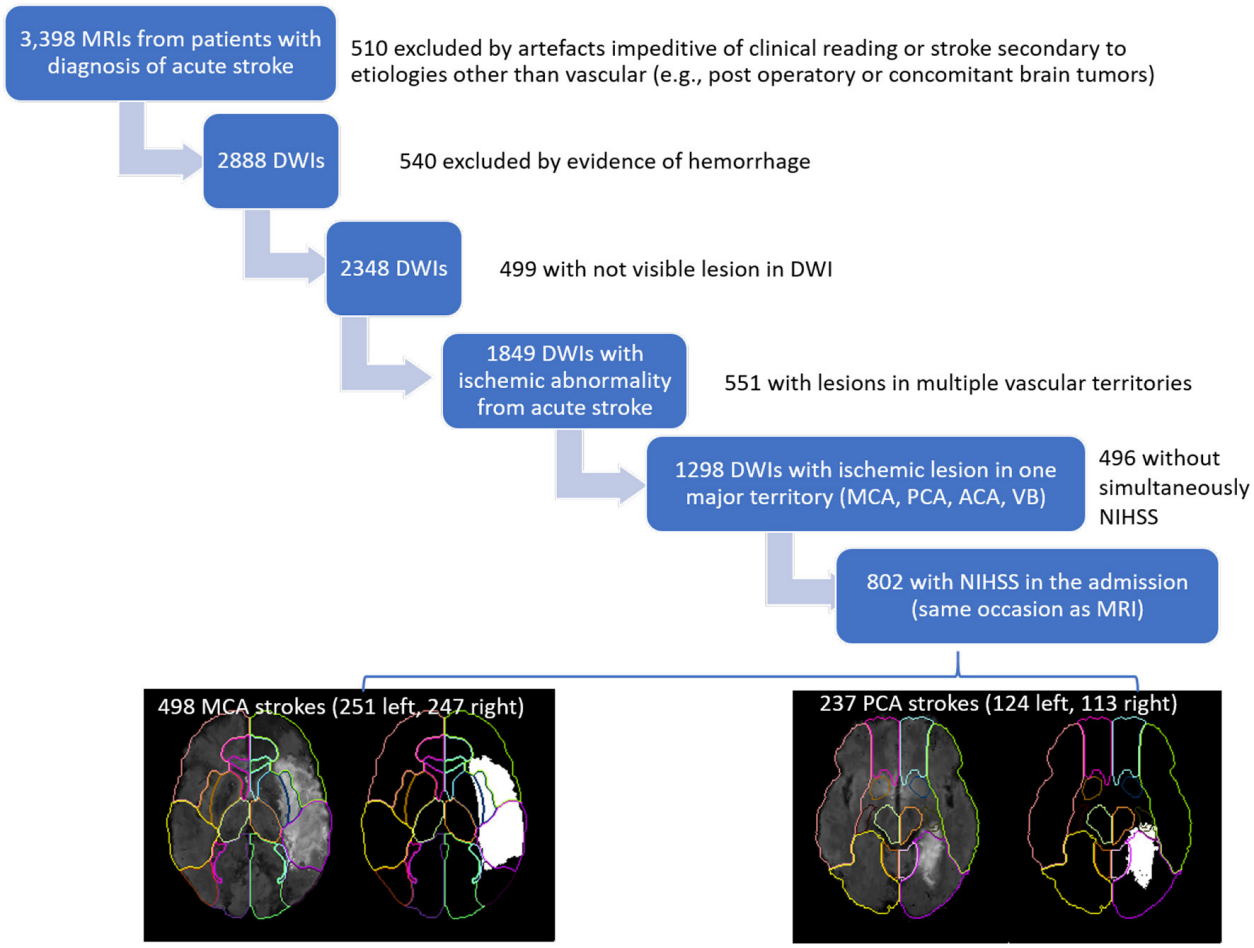
Flowchart of data inclusion.

This study included MRIs of patients admitted to the Comprehensive Stroke Center at Johns Hopkins Hospital with the clinical diagnosis of ischemic stroke, between 2009 and 2019 (Flowchart for data inclusion in Figure 1). It utilizes data from an anonymized dataset, created under waiver of informed consent (IRB00228775). We have complied with all relevant ethical regulations and the guidelines of the Johns Hopkins Institutional Review Board, that approved the present study (IRB00290649).

From the 2,888 DWIs quality-controlled for clinical analysis, 1,849 DWIs showed lesions classified by a neuroradiologist as result of acute or early subacute ischemic stroke, with no evidence of hemorrhage. From those, we included 802 individuals who had NIHSS recorded at admission, in the same occasion as the MRI, and at least 90% of the infarct lesion constrained to a single vascular territory (MCA, PCA, ACA, vertebrobasilar). Note that the stringent inclusion criteria might lead to unascertained (although inevitable) bias in the analysis. The present study focuses on the largest groups of lesions, affecting the MCA (*n* = 498) and PCA (*n* = 237) territories. The summary of demographics and lesion profiles is in Table 1. The lesion core was defined in DWI, in combination with the Apparent Diffusion Coefficient maps (ADC) by two experienced evaluators and was revised by a neuroradiologist until reaching a final decision by consensus. Further details are in our previous publication (16).

In an initial exploratory analysis, we examined differences in groups of patients with left or right stroke regarding to lesion volume, NIHSS and other covariates (age, sex, race). We used t-test for continuous variables and chi-square tests for categorical variables. We then used generalized linear models to test the effects of lesion side, volume, patient's age, sex, and race on NIHSS. We used Akaike information criterion (AIC) to evaluate the impact of covariates or of their interactions in the models. We looked at the whole sample as well as in the sample stratified by lesion location (MCA or PCA) and by NIHSS [≤5 or >5; as NIHSS ≤ 5, considered “mild stroke”, are most strongly associated with good prognosis and short hospital discharge to home (17, 18)]. We additionally stratified the MCA strokes by vascular “subdivision” (superior: lesions with more than 75% of volume in the frontal areas vs. inferior: lesions with more than 75% of volume in the temporo-parietal areas), by possible etiology (atherosclerotic or cardiogenic thrombotic), and tested the effect of ASPECTS (19) in the general linear models, in place of the volumes. The statistical analysis was performed with R.

## 3. Results

**Figure 2:**
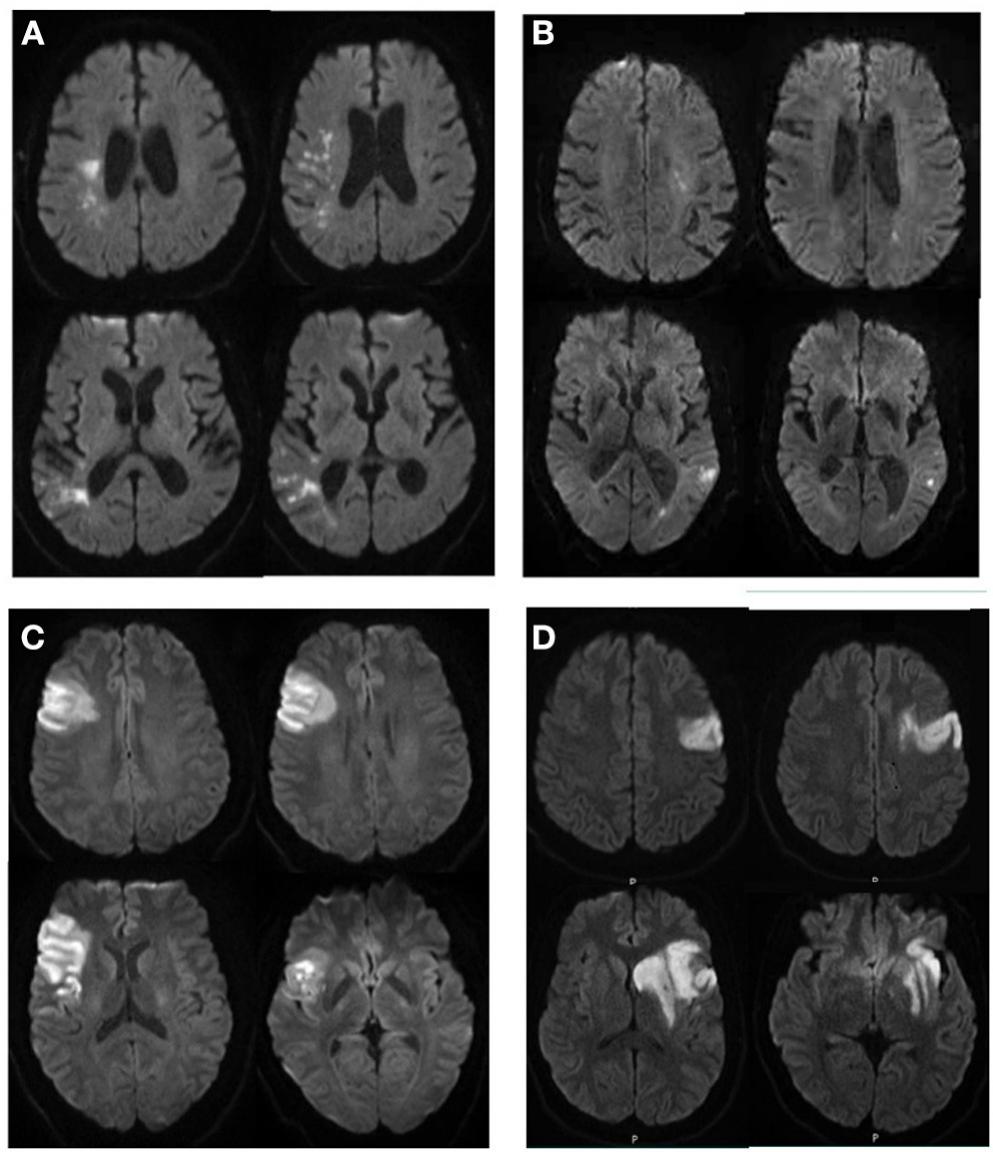
Illustrative cases of patients with right **(A,C)** and left **(B,D)** strokes, with similar NIHSS and very different infarct volumes **(A,B)**; or similar infarct volumes and very different NIHSS **(C,D)**. **(A)** 76 year-old man, NIHSS = 4, infarct volume of 11.9 cc; **(B)** 84 year-old man, NIHSS = 5, infarct volume of 2.2 cc; **(C)** 34 year-old man, NIHSS = 6, infarct volume of 39 cc; **(D)** 35 year-old man, NIHSS=10, infarct volume of 38 cc.

As shown in Table 1, there were no significant differences between left and right strokes in terms of patient's age, sex, and race. The distribution of these variables was similar in left and right strokes. Patients with left MCA strokes had significantly higher NIHSS than those with right MCA strokes. This difference was not significant in patients with PCA strokes. This left hemisphere bias is illustrated in Figure 2. In addition, right MCA strokes tended to be larger than the right counterparts (although not significantly at *p* < 0.05). This tendency persisted after stratification for NIHSS (≤5 or >5) as shown in Supplementary Table 2, stroke etiology (atherosclerotic or cardioembolic), and in strokes affecting the inferior MCA area, but not the superior (Supplementary Table 4). Note however, that the sample size of superior MCA strokes and cardioembolic strokes is small, and these results must be interpreted with caution.

The initial linear models to assess the effects of covariates in NIHSS included stroke side, volume, patient's age, sex, race, and interactions between stroke side and volume. As the effect of race was not significant in any model, race was further excluded from the analysis. Patient's sex and interactions between stroke side and volume were marginally associated to NIHSS (*p*-value for sex = 0.034; *p*-value for the interaction between side and volume = 0.03), only when considering the whole sample. The models with or without these covariates were equivalent (both showed AIC = 4,753). Therefore, sex and interaction between stroke volume and side were also excluded from further models. In summary, the final models used age, lesion side, and volume as predictors. They revealed that in patients with MCA strokes, and not in those with PCA strokes, NIHSS score is affected by the infarct side (*p*-value for infarct side = 0.00491) even after controlling for stroke volume and patient's age, as shown Table 2 and Supplementary Table 3. This effect was driven by the more severe strokes (NIHSS>5). In addition, stroke volume and patient's age significantly correlated with NIHSS.

**Table 2:** *P*-values for the generalized linear models and covariates of the models to predict the NIHSS.

	**All**			**MCA**			**PCA**		
	**Total**	**NIHSS < =5**	**NIHSS>5**	**Total**	**NIHSS < =5**	**NIHSS>5**	**Total**	**NIHSS < =5**	**NIHSS>5**
Sample	802	527	275	498	280	218	237	197	40
Intercept	0.7071	7.12E-07	3.81E-07	0.7844	0.01365	1.54E-06	0.0167	2.31E-06	0.0532
Lesion volume	<2.2e-16	0.00167	2.00E-16	2.00E-16	0.00384	7.67E-15	0.1192	0.217	0.597
Lesion side	0.00667	0.78265	0.00728	0.00491	0.1007	0.0117	0.4844	0.37	0.5479
Age	6.92E-08	0.22814	0.00817	1.10E-06	0.01438	0.0209	0.3374	0.254	0.1015
Model	<2.2e-16	0.0127	<2.2e-16	<2.2e-16	0.00175	7.44E-14	0.2523	0.394	0.2728

Within the MCA strokes, the models controlled for age and stroke volumes showed that NIHSS score is affected by the infarct side in inferior MCA infarcts and in atherosclerotic strokes (Supplementary Table 4). However, we note again that sample size of superior MCA infarcts and cardioembolic strokes is small, limiting the power of this analysis. NIHSS score was still affected by the infarct side when ASPECTS—a metric of direct clinical relevance—was considered in the place of infarct volume (Supplementary Table 5). This is not surprising as ASPECTS and infarct volumes are highly correlate between themselves (*r* = 0.72, in our sample) and with NIHSS score (*r* = 0.59 and 0.55, respectively). As happened when we categorized by NHISS scores, this effect was driven by the most severe strokes (ASPECTS < 8).

## 4. Discussion

This study evaluated the association between NIHSS score and lesion volume by vascular territory, specifically the MCA and PCA territories. We confirmed our hypothesis that right MCA ischemic strokes are larger than left MCA ischemic strokes, especially for higher NIHSS scores. We also confirmed that the NIHSS significantly depends on the infarct side of MCA strokes, after correcting for lesion volume and other covariates. Although generally in agreement with previous studies (8, 11, 12), this study was substantially larger than previous studies (*n* = 153–312), and evaluated the bias for separate vascular territories. We also controlled for age and sex, as both variables may be differential associated with infarct volume.

Previous authors have explained the hemispheric bias as a reflection of greater points given for language deficits (typically left hemisphere symptom) than hemispatial neglect (typically thought of as right hemisphere symptom). Gottesman et al. found supplementing the NIHSS with more points for neglect (assessed with line cancellation and visual extinction) could correct the bias. This additive approach would require 2–3 min but could potentially correct the left hemisphere bias (12). This bias could be corrected in other ways (e.g., eliminating orientation and commands), but this approach would yield a less complete neurological exam. It is important to recognize that the NIHSS does not capture right cortical dysfunction. Other right, mostly MCA, cortical functions include empathy (20, 21), recognition and expression of affective prosody [tone of voice to convey emotions (22, 23), recognition of facial expression (24), awareness of deficits (25), integration of information (getting the “big picture”) (26, 27), understanding humor, and metaphor (28, 29), etc.]. However, these cortical functions are more difficult to assess reliably at bedside.

Limitations of the study include the fact that we do not have reliable information about handedness of the patients. NIHSS bias could potentially be modified in non-right-handed people, but probably does not systematically modify scores more in left hemisphere or right hemisphere stroke [as there are cases of both right stroke aphasia and left stroke severe neglect in left-handed patients (30)]. We also do not have reliable information about the time of symptoms onset for many patients, but most of the scans were 6 h after symptoms (in the patients with time of onset recorded) and therefore the likelihood of significant changes in the stroke volumes based on timing is small (31).

Despite the study's limitations, the findings may have implications for future research protocols and clinical practices that utilize the NIHSS. Some treatment protocols (e.g., involving endovascular therapy) have excluded patients with low NIHSS scores. As such, patients with large volume right hemisphere stroke and low NIHSS scores (e.g., right temporal strokes that spare motor functions) may be under-treated. These patients may be left with disabling deficits that substantially impede social function and human relationships, such as failure to empathize, understand emotional tone of voice or facial expression or humor (32). Likewise, use of a “diffusion-clinical mismatch” (33–35) that uses an NIHSS score in comparison the volume of ischemia on DWI, has been advocated for thrombectomy up to 24 h post-onset of stroke. Because clinical deficits reflect volume of hypoperfusion more than volume of DWI infarct (36–38), patients with right inferior division MCA strokes are likely not to meet the clinical criteria for this important intervention but are likely to have disabling sequelae. Thus, the NIHSS alone may not be optimal for determining the lower limits of stroke treatment eligibility, specifically for right MCA stroke patients.

## Data Availability Statement

The original contributions presented in the study are publicly available. This data can be found here: Zenodo, https://zenodo.org/, 10.5281/zenodo.5722286 (39).

## Ethics Statement

The studies involving human participants were reviewed and approved by the Johns Hopkins Internal Review Board. Written informed consent for participation was not required for this study in accordance with the national legislation and the institutional requirements.

## Author Contributions

EV collected the data and drafted the work. GK and AF collected and analyzed the data. MS significantly reviewed the draft. AH and AF conceived and designed the study, interpreted the data, and drafted the work. All authors contributed to the article and approved the submitted version.

## Funding

This research was supported in part by the National Institute of Deaf and Communication Disorders, NIDCD, through R01 DC05375, R01 DC015466, and P50 DC014664 (AH, EV, MS, AF).

## Conflict of Interest

The authors declare that the research was conducted in the absence of any commercial or financial relationships that could be construed as a potential conflict of interest.

## Publisher's Note

All claims expressed in this article are solely those of the authors and do not necessarily represent those of their affiliated organizations, or those of the publisher, the editors and the reviewers. Any product that may be evaluated in this article, or claim that may be made by its manufacturer, is not guaranteed or endorsed by the publisher.
