# Supplementary material for: Left Hemisphere Bias of NIH Stroke Scale Is Most Severe for Middle Cerebral Artery Strokes
Source: Front Neurol. 2022 Jun 14;13:912782. doi: 10.3389/fneur.2022.912782 (PMC9237381; doi:10.3389/fneur.2022.912782)
Supplement: Supplementary file 1 [file Table_1.docx]

**Supplementary Tables**

|  | Left | Right |
| --- | --- | --- |
| 1a Level of consciousness (3) | X | X |
| 1b Level of consciousness – Questions (2) | X |  |
| 1c Level of consciousness – Commands (2) | X |  |
| 2 Best gaze (2) | X | X |
| 3 Visual (3) | X | X |
| 4 Facial palsy (3) | X | X |
| 5 Motor arm (4) | X | X |
| 6 Motor leg (4) | X | X |
| 7 Limb ataxia (2) | X | X |
| 8 Sensory (2) | X | X |
| 9 Best language (3) | X |  |
| 10 Dysarthria (2) | X | X |
| 11 Extinction and inattention (2) | X | X |

**Table 1.** Functions evaluated (points attributed) in NIHSS, and the expected dominance of such functions according to brain hemisphere (left or right).

|  | | **lesion side** | **NIHSS** | **lesion volume (cc)** | **Age (years)** | **Sex(%F/%M)** | **Race (%aa/%c/%other)** |
| --- | --- | --- | --- | --- | --- | --- | --- |
|  |  | Left (n=271) | 2.1 (1.6) | 8 (21.7) | 60.1 (13.2) | 43.5/56.5 | 60.1/36.9/3 |
|  | **NIHSS<=5** | Right (n=256) | 2.1 (1.6) | 8 (15) | 62.4 (13.5) | 41.8/58.2 | 60.2/36.7/3.1 |
| **All** |  | Left (n=149) | 12.6 (6.3) | 49.6 (73.8) | 64.9 (14.1) | 49/51 | 57.7/32.2/10.1 |
|  | **NIHSS>5** | Right (n=126) | 11.4 (4.8) | 59.6 (79.8) | 63.9 (15.8) | 57.1/42.9 | 65.1/27/7.9 |
|  |  | Left (n=136) | 2.4 (1.7) | 9.5 (16.6) | 61.1 (13.5) | 46.3/53.7 | 51.5/45.6/2.9 |
|  | **NIHSS<=5** | Right (n=144) | 2.0 (1.6) | 10.6 (28.4) | 61.1 (14.1) | 40.3/59.7 | 57.6/38.9/3.5 |
| **MCA** |  | Left (n=115) | 13.6 (6.5) | 61.4 (79.3) | 63.9 (14.4) | 52.2/47.8 | 52.2/36.5/11.3 |
|  | **NIHSS>5** | Right (n=103) | 12.2 (4.9) | 71.5 (83.5) | 64.4 (16.0) | 55.3/44.7 | 64.1/29.1/6.8 |
|  |  | Left (n=101) | 1.9 (1.4) | 6.1 (12.2) | 59.2 (13.1) | 38.9/61.4 | 73.3/24.8/2 |
|  | **NIHSS<=5** | Right (n=96) | 2.1 (1.5) | 6.7 (13.3) | 64.2 (12.2) | 42.7/57.3 | 64.6/33.3/2.1 |
| **PCA** |  | Left (n=23) | 8.3 (3.3) | 12.2 (29.8) | 65.6 (12.7) | 47.8/52.2 | 78.3/17.4/4.3 |
|  | **NIHSS>5** | Right (n=17) | 7.5 (1.6) | 4.2 (7.7) | 61.0 (13.7) | 58.8/41.2 | 64.7/23.5/11.8 |

**Table 2.** Demographic and lesion characteristics in samples stratified by the vascular territory affected and by NIHSS. F=female, M=male, aa=African Americans, c=caucasians, others include others and unknown races. Continuous variables are represented as mean (standard deviation)

|  | | All | MCA | PCA |
| --- | --- | --- | --- | --- |
|  | volume | 0.988 / 0.045 / 21.578 | 0.94 / 0.054 / 17.346 | 0.294 / 0.188 / 1.564 |
| any NIHSS | side | -0.898 / 0.330 / -2.720 | -1.327 / 0.469 / -2.826 | -0.258 / 0.369 / -0.700 |
|  | age | 0.064 / 0.011 / 5.445 | 0.080 / 0.016 / 4.934 | 0.013 / 0.014 / 0.961 |
|  | volume | 0.167 / 0.053 / 3.160 | 0.179 / 0.061 / 2.916 | 0.153 / 0.124 / 1.239 |
| NIHSS<=5 | side | -0.037 / 0.136 / -0.276 | -0.320 / 0.194 / -1.647 | 0.187 / 0.209 / 0.898 |
|  | age | 0.006 / 0.005 / 1.207 | 0.017 / 0.007 / 2.463 | -0.009 / 0.008 / -1.145 |
|  | volume | 0.575 / 0.055 / 10.341 | 0.521 / 0.062 / 8.366 | 0.156 / 0.294 / 0.533 |
| NIHSS>5 | side | -1.571 / 0.581 / -2.704 | -1.745 / 0.685 / -2.544 | -0.539 / 0.889 / -0.607 |
|  | age | 0.052 / 0.019 / 2.665 | 0.053 / 0.023 / 2.327 | 0.056 / 0.033 / 1.681 |

**Table 3.** Estimates / Standard errors / t values for the covariates (lesion volume, lesion side and patient age) of the generalized linear models to predict the NIHSS.

|  | | **group means (standard deviation)** | | | **GLM (covariates p-values)** | | |
| --- | --- | --- | --- | --- | --- | --- | --- |
|  |  | **Left** | **Right** | **All** | **volume** | **side** | **age** |
| **MCA branch** | **inferior**  sample size lesion volume  NIHSS | 72  27626(44247)  6.06 (6.1) | 50  33843(56380)  4.56(4.5) | 122  30174(49452)  5.4(3.5) | <10e-8 | 0.035 | 0.02 |
|  | **superior**  sample size lesion volume  NIHSS | 37  11601(15402)  3.7(3.8) | 35  10519(14292)  3.8(3.5) | 72  11075(14778)  3.7(3.7) | 0.0004 | 0.2 | 0.12 |
| **etiology** | **cardioembolic** sample size lesion volume  NIHSS | 30  21170(25979)  6.4(5.7) | 24  33937(63753)  5.3(4) | 54  26604(46591)  5.9(5) | 0.012 | 0.2 | 0.5 |
|  | **atherosclerotic** | 126 | 124 | 250 | <10e-15 | 0.014 | <10e-4 |
|  | sample size |  |  |  |  |  |  |
|  | lesion volume | 2483(46887) | 36086(68282) | 30430(58637) |  |  |  |
|  | NIHSS | 5.7(5.9) | 5(5.6) | 5.4(5.7) |  |  |  |

**Table 4.** MCA infarcts stratified by the subregions affected and most likely stroke etiology. At right, the p-values for the covariates in models derived from each stratified group. Volumes are in ml.

|  | ASPECTS | side | age |
| --- | --- | --- | --- |
| ASPECTS<10 (n=268) | <10e-15 | 0.0024 | <10e-6 |
| ASPECTS>=8 (n=107) | 0.89 | 0.2 | <10e-4 |
| ASPECTS<8 (n=161) | <10e-14 | 0.0005 | 0.00018 |

**Table 5.** t-values for the covariates (ASPECTS, lesion side and patient age) of the generalized linear models to predict the NIHSS.
